# Molecular Characterization and Expression Profiles of *Sp-uchl3* and *Sp-uchl5* during Gonad Development of *Scylla paramamosain*

**DOI:** 10.3390/molecules23010213

**Published:** 2018-01-19

**Authors:** Kunhuang Han, Yanbin Dai, Ziping Zhang, Zhihua Zou, Yilei Wang

**Affiliations:** 1 Key Laboratory of Healthy Mariculture for the East China Sea, Ministry of Agriculture, Fisheries College, Jimei University, Xiamen 361021, China; hankunhuang@foxmail.com (K.H.); daiyanbin@jmu.edu.cn (Y.D.); zouzhihua@jmu.edu.cn (Z.Z.); 2 State Key Laboratory of Large Yellow Croaker Breeding, Ningde Fufa Fisheries Company Limited, Ningde 352103, China; 3 College of Animal Science, Fujian Agriculture and Forestry University, Fuzhou 350002, China; zhangziping@fafu.edu.cn

**Keywords:** *Scylla paramamosain*, *Sp-uchl3*, *Sp-uchl5*, expression profile, tissue, gonadal development

## Abstract

Ubiquitin C-terminal hydrolases (UCHLs) are a subset of deubiquitinating enzymes, and are involved in numerous physiological processes. However, the role of UCHLs during gonad development has not been studied in crustaceans. In this study, we have first cloned and analyzed expression profiling of *Sp-uchl3* and *Sp-uchl5* genes from mud crab *Scylla paramamosain*. The full-length cDNA of *Sp-uchl3* is of 1804 bp. Its expression level in the ovary was significantly higher than in other tissues (*p* < 0.01), and during gonadal development, its expression in both O1 and O5 stages was significantly higher than in the other three stages of ovaries (*p* < 0.05), while in T3 it was higher than in the former two stages of testes (*p* < 0.05). Meanwhile, the full-length cDNA of *Sp-UCHL5* is 1217 bp. The expression level in the ovary was significantly higher than in other tissues (*p* < 0.01). Its expression in ovaries was higher than in testes during gonadal development (*p* < 0.05). The expression level in the O5 stage was the highest, followed by the O3 stage in ovarian development, and with no significant difference in the testis development (*p >* 0.05). These results provide basic data showing the role of *Sp*-UCHL3 and *Sp*-UCHL5 in the gonad development of the crab.

## 1. Introduction

The green mud crab *Scylla paramamosain* is mainly distributed in the southeastern coast of China and is an important economic species in aquaculture. Due to its large size, rapid growth, delicate flavor and abundant nutrient components, the artificial cultivation of the crab has become one of the main aquaculture industries in China [1]. With the development of crab cultivation, the artificial breeding of crabs had been explored by many researchers [2]. However, the aquaculture production currently still relies heavily on wild-caught fry for farming, and the amount of wild-caught fry fluctuates greatly. Insufficient and unstable resources of natural seedlings have become the bottleneck in the health and sustainability of the development of green mud crab aquaculture [3]. Therefore, the fry production techniques related to improving spawning, breeding survival rate and decreasing the cost have become the current main tasks in the aquaculture production of the crab [4]. Thus, it is important to understand the molecular regulatory mechanisms involved in gonad development and gametogenesis in order to provide reliable hatchery technology for production of crab fry. In recent years, many researchers have carried out a lot of work on this aspect. For example, Gong et al. [5] reported that retinoid X receptor and methyl farnesoate play an important role in regulating the expression of vitellogenin mRNA and ovarian development of *S. paramamosain.* Zeng et al. [6] found that red pigment concentrating hormone (RPCH) may play important roles in the ovarian maturation by directly regulating vitellogenesis in the ovary and hepatopancreas. Bao et al. [7] found that 21 neuropeptide transcripts from the transcriptome data of female cerebral ganglia had differential expressions during various vitellogenic stages of *S. paramamosain*, which may be involved in regulating vitellogenesis and ovarian maturation. Our previous work has constructed three different normalized expressed sequence tags (ESTs)_ libraries, which were created from testis, ovary and mixed organs of *S. paramamosain*, and had identified several gonad development-related genes [8]. Furthermore, we have found 33 ovary-specific, 14 testis-specific and 34 gonad-differential transcripts from the transcriptome sequencing data of the testis and ovary of *S. paramamosain* [9]*.* Based on these results, we cloned and characterized some gonad development-related genes, such as *ubiquitin-conjugating enzyme E2 isoform 2* [8], *vasa* [10], *vitellogenin* [11], *erk2* [12], *cdc2* and *cyclin b* [13], *ubiquitin* [14] and *SUMO1* [15], and detected their expression during gonad development and gametogenesis by real-time quantitative PCR (RT-qPCR) and in-situ hybridization. The proteins encoded by these genes are important and play a regulatory role in the cell cycle and cellular growth during gonad development. These results provide the basic data for the gonad development or gametogenesis of *S. paramamosain*. 

As is known to all, the expression of proteins involved in the regulation of the cell cycle is changed dynamically to meet the physiological functions. The ubiquitin proteasome pathway (UPP), a major intracellular system for ATP-dependent extra-lysosomal protein degradation in the cell cycle, mediates the degradation of 80–85% of intracellular proteins. It consists of ubiquitin, ubiquitin-activating enzymes E1, ubiquitin-conjugating enzymes E2, ubiquitin ligases E3, proteasome and deubiquitinating enzyme [16]. Numerous studies have demonstrated that UPP plays critical roles in controlling the levels of various cellular proteins, thereby regulating basic cellular processes such as cell division and growth, apoptosis, signal transduction, DNA damage and repair, immune response, and so on [17,18,19,20,21,22]. In recent years, a growing number of reports have indicated that UPP is involved in reproductive regulation, especially in the control of meiosis, reconstruction of the chromatin structure, spermatogenesis and sperm maturation, and oocyte meiotic maturation [23,24,25,26,27].

Ubiquitin C-terminal hydrolases (UCHs), formerly known as ubiquitin carboxyl-terminal esterases [28], are deubiquitinating enzymes (DUBs) that are involved in cotranslational processing of pro-ubiquitin gene products, and catalyze the removal of peptides and small molecules (such as amines and thiol groups) from the C-terminus of ubiquitin [29]. UCHs are relatively small enzymes that are structurally defined by a signature active site bearing a catalytic triad of positionally conserved cysteine/histidine/aspartic acid residues. There are many members in the UCH superfamily: UCH-L1-5, CYLD and so on [30,31]. The UCH-L1 involvement in regulation of gonad development has been well reported. For example, UCH-L1 is abundantly expressed in Sertoli cells and oocytes in mice and it also plays a role in the sperm–oocyte interaction of the zona pellucida during fertilization [32,33]. Sun et al. [34] found that a protein (tUCH) derived from toad (*Bufo bufo gargarizans*) oocytes had the ability to hydrolyze the UCH substrate ubiquitin ethyl ester, had a high sequence homology to mammalian UCH-L1, and they furthermore showed that the protein is involved in oocyte maturation regulation through a kinetics study. Mochida et al. [35] found that UCH was expressed in the ovary and in testis of tilapia (*Oreochromis niloticus*), and localized especially in pre-vitellogenic oocytes in the ovary, implying that the enzyme activity could be important in oocyte growth. Other studies have shown that UPP system-related factors are involved in the regulation of crustacean gonad development [36]. However, the detailed role of the UCH family genes in gonad development of crustaceans has not yet been elucidated. 

In this study, the full-length cDNAs of *uchl3* and *uchl5* from *S. paramamosain* were first identified, and their expressions in different tissues and developing gonad were determined. The cloning and characterization of *Sp-uchl3* and *Sp-uchl5* transcripts will provide us with useful information to further investigate the molecular mechanism of gonad development in crabs.

## 2. Results

### 2.1. Molecular Characterization of Sp-uchl3 and Sp-uchl5

**Figure 1:**
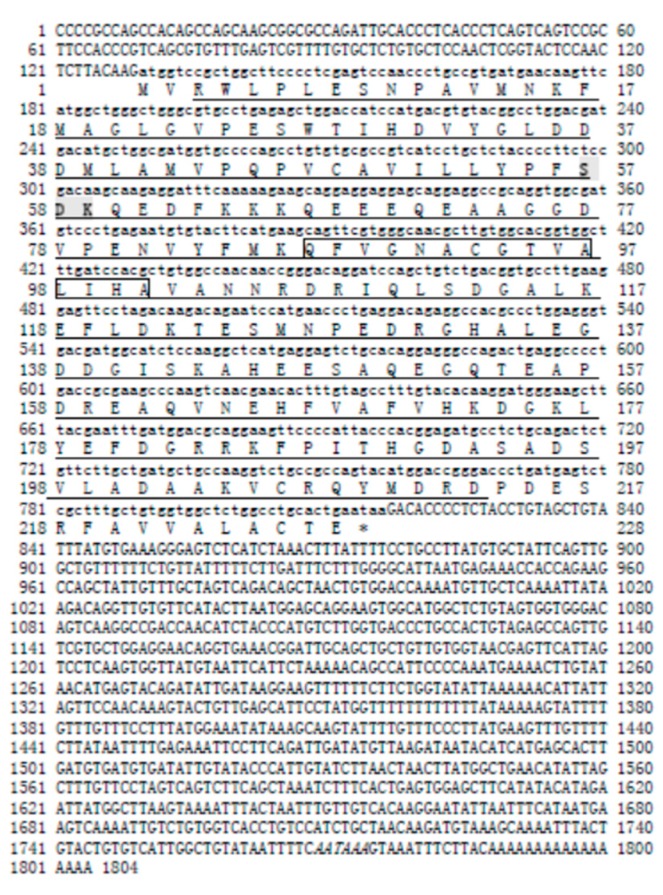
The cDNA and deduced amino acid sequence of *Sp-uchl3.* The capital bases represent the non-coding region. The small bases represent the open reading frame. The conserved sequence of the UCHL1 family is underlined, the phosphorylation site of protein kinase C is indicated by shadow and bold (S^57^–K^59^), the active site of ubiquitin carboxyl-terminal hydrolases family 1 is in the box (Q^87^–A^103^), and the polyadenylation signal (AATAA) is marked by italics.

**Figure 2:**
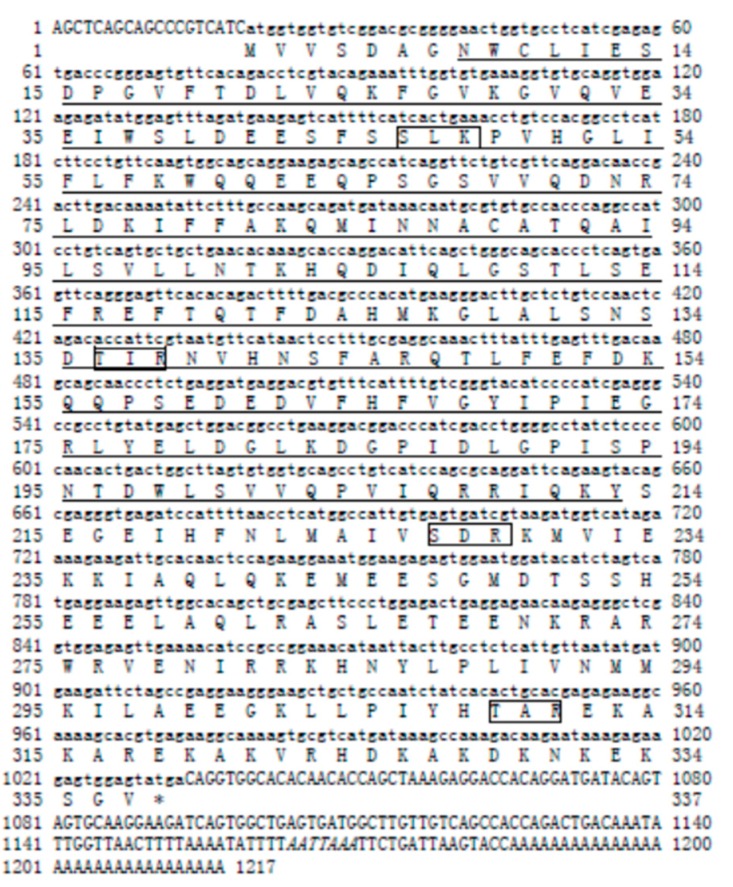
cDNA sequence and deduced amino acid sequence of *Sp-uchl5.* The capital bases represent the non-coding region, the small bases represent the open reading frame. The conserved sequence of the UCHL1 family is underlined, the 4 phosphorylation sites of protein kinase C are indicated in the box, and the polyadenylation signal (AATTAAA) is enclosed by italics.

The full-length *Sp-uchl3* cDNA is 1804 bp, containing 129 bp in the 5′-untranslated region (UTR), 687 bp in the ORF, and 988 bp in the 3′-UTR with a poly(A) tail. The cDNA sequence and deduced amino acid sequence have been submitted to the NCBI GenBank (accession No. FJ800570). The ORF encodes a polypeptide of 228 amino acids with a predicted molecular mass of 25.35 kDa and a theoretical pI of 4.64. A polyadenylation signal (AATAAA) is located 13 bp upstream of poly(A) tail (Figure 1). The results of the k-nearest neighbor (k-NN) prediction by PSORTII revealed that *Sp*-UCHL3 protein may mainly locate in the cytoplasm (65.2%). Sequence analysis revealed that it has a protein kinase C phosphorylation site (S^57^–K^59^), three casein kinase II phosphorylation sites (T28–D31, S148–E151, T189–D192), three N-myristoylation sites (G^22^–W^27^, G^90^–T^95^, G^140^–H^14^5), one amidation site (D^181^–R^184^) and an active site of ubiquitin C-terminal hydrolases family 1 (Q^87^–A^103^) (Figure 1). The full-length *Sp*-*UCHL5* cDNA is 1217 bp, including 19 bp of 5′-UTR, 1014 bp of ORF and 184 bp of 3′-UTR with a poly(A) tail (Figure 2). The sequence of *Sp*-*uchl5* has been deposited in the GenBank database under accession no. FJ595021. The ORF encodes a polypeptide of 337 amino acids, with a predicted molecular weight of 38.69 kDa and theoretical pI of 5.87. The results of the k-NN prediction by PSORTII revealed that the *Sp*-UCHL5 protein may mainly locate in the cytoplasm (60.9%). The deduced protein sequence contains four phosphorylation sites of protein kinase C (S^46^–K^48^, T^136^–R^138^, S^227^–R^229^, T^309^–R^311^), ten phosphorylation sites of casein kinase II (S^38^–E^41^, T^111^–E^114^, S^132^–D^135^, T^148^–E^151^, S^158^–E^161^, S^214^–E^217^, S^247^–D^250^, S^252^–E^255^, S^253^–E^256^, T^309^–E^312^) and three *N*-myristoylation sites (G^109^–E^114^, G^128^–N^133^, G^248^–S^253^) (Figure 2). 

### 2.2. Prediction of Sp-UCHL3 and Sp-UCHL5 Three-Dimensional (3D) Structure

**Figure 3:**
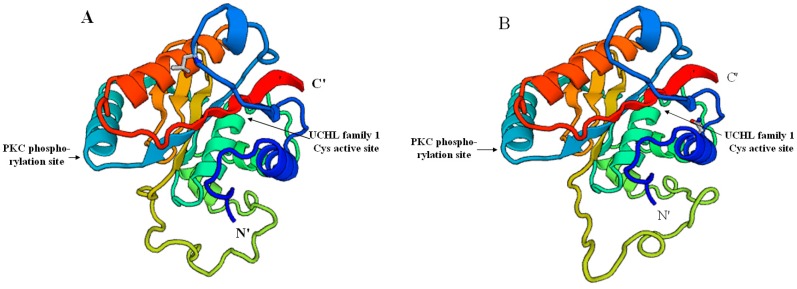
The predicted three-dimensional structure of *Sp*-UCHL3 (**A**) and *Pc-*UCHL3 (**B**).

**Figure 4:**
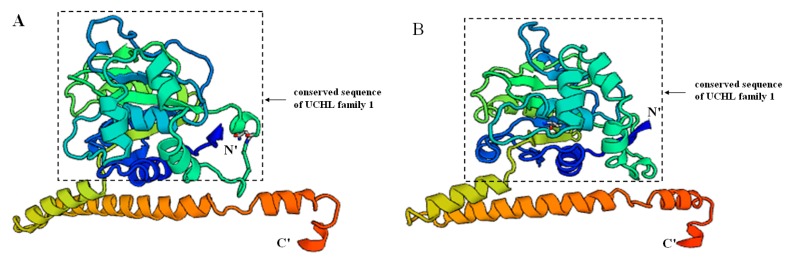
The predicted three-dimensional structure of *Sp*-UCHL5 (**A**) and *Ha*-UCHL5 (**B**).

To understand the different structures of UCHL3 between *S. paramamosain* and its closely related species *Procambarus clarkii* (GenBank: ANI85922.1), we used the template (PDB code: 1uch1A) at the SWISS-MODEL server by a homology modeling method to predict their three-dimensional structures (Figure 3). *Sp*-UCHL3 shared 52.21% sequence identity to the template, while *Pc-*UCHL3 shared 56.19%. The structure of *Sp*-UCHL3 (Figure 3A) was based on the 226 amino acid residues (V^2^–T^22^^7^) of the mature protein, and the *Pc-*UCHL3 (Figure 3B) was based on the 226 amino acid residues (V^2^–S^227^). Both *Sp*-UCHL3 and *Pc-*UCHL3 were mainly composed of nine alpha helices, seven beta sheets and several random coils, which contained the protein kinase C (PKC) phosphorylation site and the active site of ubiquitin C-terminal hydrolases family 1. The shape of a random coil located in the seventh alpha helix and the fourth beta sheet was their biggest difference. Meanwhile, the three-dimensional structures of UCHL5 (Figure 4) in *S. paramamosain* and its closely related species *Hyalella azteca* (GenBank: XP_018019441.1), according to the template (PDB code: 3ihrA): *Sp*-UCHL5 shared 64.15% sequence identity to the template, while *Ha*-UCHL5 shared 57.14%. The structure of *Sp*-UCHL5 (Figure 4A) was based on the 319 amino acid residues (V^3^–K^3^^21^) of the mature protein, while the *Ha*-UCHL5 (Figure 4B) was based on the 312 amino acid residues (A^4^–A^315^). Both of *Sp*-UCHL5 and *Ha*-UCHL5 consisted of thirteen alpha helices, six beta sheets and several random coils, while the conserved sequence of UCHL family 1 (N^8^–Y^213^) was located between the first beta sheet and the ninth alpha helix. The biggest difference between the *Sp*-UCHL5 and *Ha*-UCHL5 was the shape of the random coil located in the sixth alpha helix and the third beta sheet.

### 2.3. Phylogenetic and Homology Analyses of Sp-UCHL3 and Sp-UCHL5

**Figure 5:**
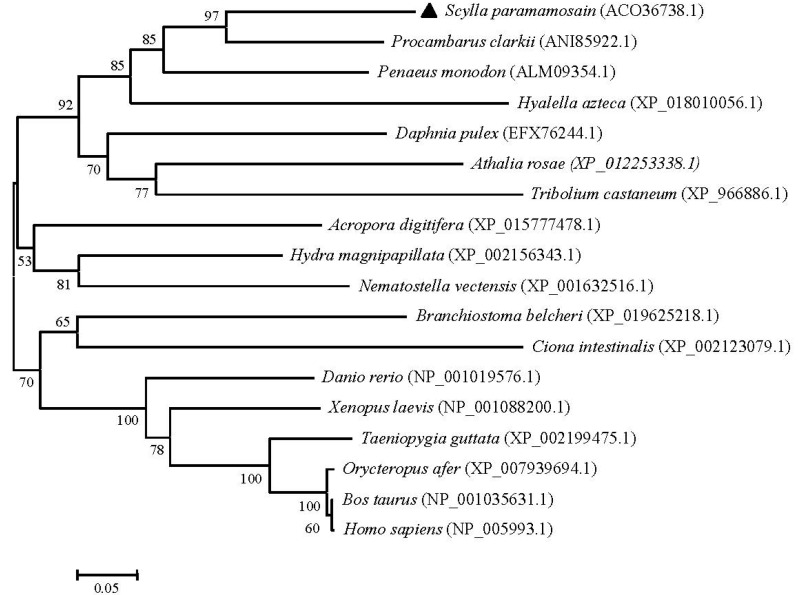
Phylogenetic tree of the UCHL3 amino acid sequences between *S. paramamosain* and other species. The GenBank codes of species are described in brackets. The *Sp-*UCHL3 is marked by black triangle. The numbers near the node mean bootstrap values of 1000 replicates.

**Figure 6:**
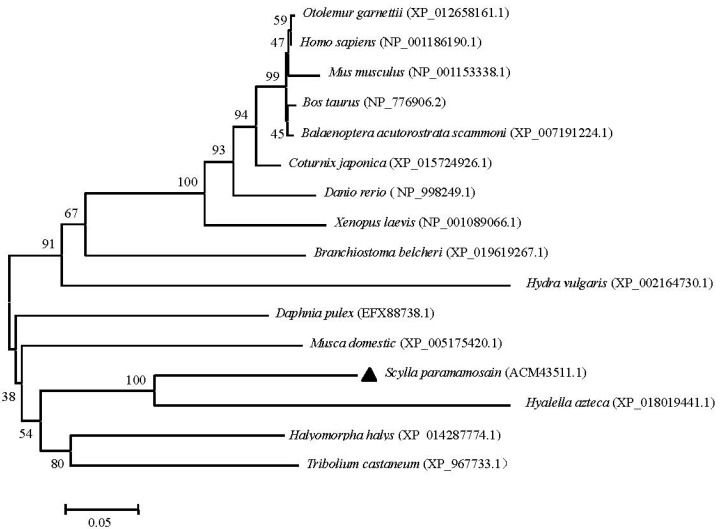
Phylogenetic tree of the UCHL5 amino acid sequences between *S. paramamosain* and other species. The GenBank codes of species are described in brackets. The *Sp-*UCHL3 is marked by black triangle. The numbers near the node mean bootstrap values of 1000 replicates.

To examine the relationships of UCHL3 and UCHL5 among various animal species, the phylogenetic trees were generated separately by using the MEGA 4.0 neighbor-joining method based on putative amino acid sequences of green mud crab and the other known UCHL3 and UCHL5 retrieved from GenBank. The results showed that the selected UCHL3 sequences were clustered into two groups: chordata and invertebrate. The chordata group consisted of two subgroups: the first subgroup included vertebrate-containing fish (*Danio rerio*), amphibians (*Xenopus laevis*), birds (*Taeniopygia guttata*) and mammals (*Orycteropus afer*, *Bos taurus* and *Homo sapiens*), while the second subgroup included Tunicata (*Ciona intestinalis*) and Cephalochordata (*Branchiostoma belcheri*). Furthermore, the invertebrate group consisted of two subgroups: cnidaria and arthropods. Obviously, *Sp*-UCHL3 belonged to the arthropod group, first being clustered with *Procambarus clarkii*, and next with *Penaeus monodon* (Figure 5). Meanwhile, phylogenetic analysis of the selected UCHL5 amino acid sequences revealed that UCHL5 proteins were formed into two main clusters. Vertebrates, including mammals, birds and amphibians formed a subcluster, and invertebrates including *S. paramamosain*, *Hyalella azteca* and *Tribolium castaneum* formed the other subcluster. Interestingly, *Hydra vulgris* was grouped to the vertebrates cluster (Figure 6).

### 2.4. Tissue Distribution of Sp-uchl3 and Sp-uchl5 Transcripts in Female Crab

**Figure 7:**
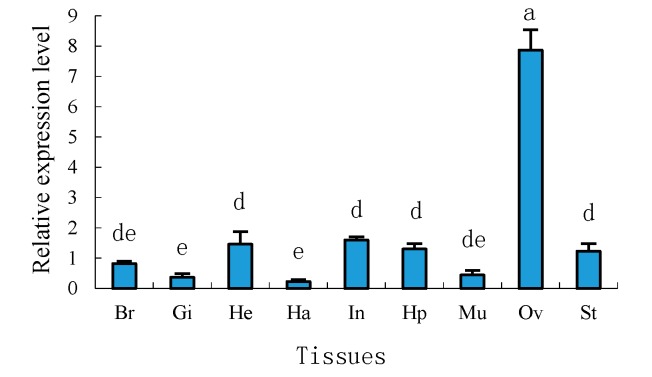
Distribution pattern of *Sp-uchl3* in various tissues. Br: brain; Gi: gill; He: heart; Ha: haemolymph; In: intestine; Hp: hepatopancreas; Mu: muscle; Ov: ovary; St: stomach. The lowercase letters above error bars signify difference as follows: the same letters are not significantly different (*p >* 0.05), the adjacent letters are significantly different (*p* < 0.05), and the intervallic letters are extremely significantly different (*p* < 0.01).

**Figure 8:**
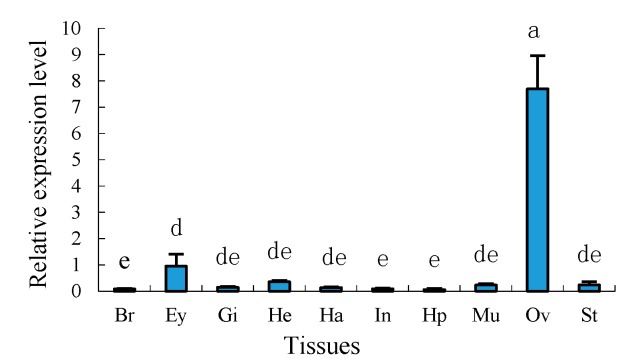
Distribution pattern of *Sp-uchl5* in various tissues. Br: brain; Gi: gill; He: heart; Ha: haemolymph; In: intestine; Hp: hepatopancreas; Mu: muscle; Ov: ovary; St: stomach. The lowercase letters above error bars signify difference as follows: The same letters are not significantly different (*p >* 0.05), the adjacent letters are significantly different (*p* < 0.05), and the intervallic letters are extremely significantly different (*p* < 0.01).

Tissue distributions of mRNA expression of *Sp-uchl3* and *Sp-uchl5* in female crab were examined by quantitative real time polymerase chain reaction (RT-qPCR) using 18S rRNA as an internal control gene. The results showed that the *Sp-uchl3* (Figure 7) and *Sp-uchl5* (Figure 8) were differentially expressed in the analyzed tissues including brain, gill, heart, haemocytes, intestine, hepatopancreas, muscle, ovary and stomach. However, their expression levels in the ovary were significantly higher than in other examined tissues (*p* < 0.01).

### 2.5. Expression Profiles of Sp-uchl3 and Sp-uchl5 Transcripts in the Different Stages of Gonad Development

**Figure 9:**
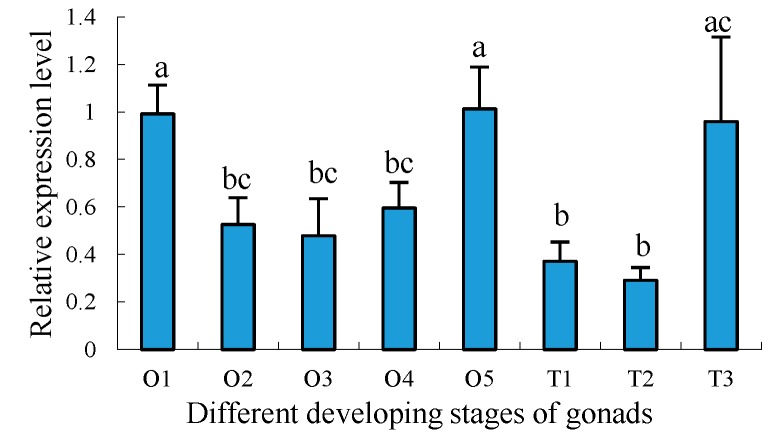
Distribution pattern of *Sp-uchl3* in different developing stages of gonad. O: ovary, T: testis, number: stage of development. Different lowercase letters above error bars indicate significantly differential expression (*p* < 0.05).

**Figure 10:**
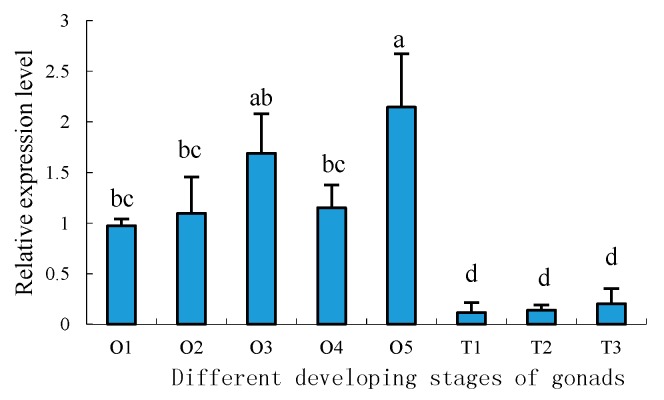
Distribution pattern of *Sp-uchl5* in different developing stages of gonad. O: ovary, T: testis, number: stage of development. The lowercase letters above error bars signify difference as follows: The same letters are not significantly different (*p >* 0.05), the adjacent letters are significantly different (*p* < 0.05), and the intervallic letters are extremely significantly different (*p* < 0.01).

To further understand the developmental expression profiles of *Sp-uchl3* and *Sp-uchl5* in different gonad developing stages, RT-qPCR was employed to compare their relative expression levels. The results showed that the expression of *Sp-uchl3* mRNA was the highest in the O5 stage, followed by O1 and T3 stages, and the lowest in the T2 stage. The expression levels of *Sp-uchl3* in both O1 and O5 stages were significantly higher than in the O2, O3 and O4 stages of ovaries (*p* < 0.05), and its expression in T3 was significantly higher than in the T1 and T2 stages of testes (*p* < 0.05) (Figure 9). Meanwhile, the *Sp-uchl5* expression level in ovaries was higher than in testes (*p* < 0.05). The expression level in the O5 stage was the highest, followed by the O3 stage during ovarian development, and there was no significant difference between the O3 and O5 stages, while their expression levels were significantly higher than in testes (*p* < 0.01). However, the expression level in the O5 stage was much higher than in the O1, O2 and O4 stages (*p* < 0.05). Moreover, the expression levels in the three stages of testes development were not significantly different from each other (*p >* 0.05) (Figure 10). 

## 3. Discussion

The UPP is the major proteolytic pathway for specific intracellular protein degradation in eukaryotes. Its function is implicated in numerous physiological processes including cell cycle regulation, signal transduction, antigen presenting, apoptosis and so on [18,19,20,37,38,39]. UCHs are important deubiquitinating enzymes which participate in the proteolytic process of polymeric ubiquitin to promote the ubiquitin recirculation process [28,40,41,42]. They are important for the generation of monomeric ubiquitin, and are the active components of the eukaryotic ubiquitin-dependent proteolytic system [43,44]. 

This is the first report of cloning and expression profiling of UCHs in crustaceans. The full-length *Sp-uchl3* and *Sp-uchl5* cDNA sequences from *S. paramamosain* were cloned successfully, and sequence analysis of these two genes showed that both of them contain UCH family 1 conserved sequences such as cysteine, histidine and aspartate, which are important for the substrate specificity of the enzymes [45,46,47]. The N-terminal amino acid sequence residue domain of *Sp*-UCHL3 from 87 aa to 103 aa is an active site of UCH family 1. Similarly, *Sp*-UCHL5 contains a longer domain structure of UCH family 1 from 8 aa to 213 aa. UCHL1 and UCHL3 are the two most predominant isozymes in UCHs, share more than 50% sequence identity, and hydrolyze C-terminal esters and amides of ubiquitin [48,49]; moreover, UCHL5 plays a direct role in recycling ubiquitin from proteasomal degradation [50]. In the 3D-structure analysis, both *Sp*-UCHL3 and *Pc-*UCHL3 contained a PKC phosphorylation site and active site of UCH family 1; their similar structures may be responsible for their analogous functions. Meanwhile, although the structure of *Sp*-UCHL5 and *Ha*-UCHL5 share a similar conserved sequence of UCHL family 1, there were some differences in this conserved sequence compared with the two closely related species of UCHL3, which may relate to their different evolutionary status. The phylogenetic tree revealed that the both *Sp*-UCHL3 and *Sp*-UCHL5 belonged to an independent subgroup with other arthropods individually, which suggested that the evolution of these two proteins is consistent with the evolution of their species. Most UCHs can catalyze the peptide or isopeptide bond at the ubiquitin C-terminus, rather than produce monomeric ubiquitin from substrate protein conjugates or disassemble polyubiquitin chains [51]. We found that the *Sp*-UCHL3 contains the active-site crossover loop of UCHL family 1, which allows binding ubiquitins attached to an unfolded peptide [47], and *Sp*-UCHL5 may be relevant to its catalytic activity and substrate specificity for cleaving isopeptide ubiquitin chains [52]. 

UCHL1 and UCHL3 have been reported to play an important regulatory role in oocyte maturation, but UCHL5 is rarely known [53,54]. In this study, we have found that the expression levels of *Sp-uchl3* and *Sp-uchl5* mRNAs in the ovary were remarkably higher than in other tissues examined. This phenomenon was inconsistent with the report that *Sp-ub* was ubiquitous in examined tissues [14], but coincided with the tissue expression profiles of *Sp-cdc2* and *Sp-cycb* [13]. Ubiquitous expression of ubiquitin may be because ubiquitin is an important regulatory factor for many critical biological processes [55]. Furthermore, cdc2 and cycb are two important components of the maturation-promoting factor (MPF), which is a key regulator of controlling the G2/M phase transition in the meiotic maturation of oocytes and spermatocytes in animals [56]. Furthermore, the expression patterns in different ovary development stages showed that both *Sp-uchl3* and *Sp-uchl5* were highest in the O5 stage, and the second-highest expression level of *Sp-uchl3* was in the O1 stage and significantly higher than in the O2 to O4 stages. However, there was no significant difference in *Sp-uchl5* expression from the O1 to O4 stages. The former research reported that the activity of UCHs could influence cytokinesis on association with the oocyte cortex and meiotic spindle during oocyte maturation [57]. High mRNA expression of UCHs also was detected in the GV and MII stages of pig oocytes [58]. In fish [35] and amphibians [59], the UCHL1 mRNA was highly expressed in the ovary, especially in previtellogenic oocytes, suggesting that it may be important in oocyte growth and maturation. In the toad (*Bufo bufo* gargarizans), Sun et al. [34] found that tUCH plays an important role in regulating the germinal vesicle breakdown (GVBD) in oocyte maturation. In the mouse, Sekiguchi et al. [33] stated that UCH-L3 was mainly detected in the cytoplasm of mouse ovaries and strongly expressed throughout all stages of oogenesis, which suggested that it participated in the maturation of the oocyte. The rate of cell division in the crab ovary is significantly faster than in other tissues, especially in the process of mud crab ovary maturation. The diameter and number of oocytes increase rapidly [60], and the yolk is quickly accumulated in this stage, which needs a lot of enzymes to regulate intracellular proteins’ constant synthesis and degradation in order to meet the requirements of completing cell division [61,62]. UCHs are important deubiquitinating enzymes which are involved in the regulation of numerous biological processes such as cell proliferation and growth, development, and transcriptional regulation [37]. Therefore, the expression patterns of *Sp-UCHL3* and *Sp-UCHL5* in different tissues and ovarian development suggest that they play important roles in cellular proteins’ degradation during oogenesis and ovarian maturation.

Previous studies have demonstrated that the deubiquitinating enzymes play an essential regulatory contribution in spermatogenesis [63]. In humans, UCHL3 plays a pivotal role in the differentiation of spermatocytes into spermatids [64]. In mice, the researchers reported that *UCHL3* and *UCHL5* mRNAs are mainly expressed in spermatocytes and spermatids during spermatogenesis, and further studies had shown that *UCHL3* may play an important role in meiosis that differentiates spermatocytes into spermatozoa, while *UCHL1* is required for normal spermatogenesis and sperm quality control [65,66,67]. In the present study, *Sp-UCHL3* mRNA expression in T3 was significantly higher than in the T1 and T2 stages (*p* < 0.05), while *Sp-UCHL5* mRNA expressions in the three stages of testes development were not significantly different from each other (*p* > 0.05), indicating that *Sp-*UCHL3 and *Sp-*UCHL5 have different aspects in regulating mechanism of testis development. Furthermore, *Sp-*UCHL3—rather than *Sp-UCHL5—*may play a dominant important role in sperm maturation, whose phenomenon coincides with the former reports of mice [48,65]. 

## 4. Materials and Methods

### 4.1. Animals and Tissue Collection

*S. paramamosain* at different stages of the ovary and testis development was purchased from a local market of Jimei, Xiamen, China. Various tissues including ovary, muscle, heart, gill, brain, stomach, hepatopancreas, intestine and haemocytes from the mature female crab were immediately frozen in liquid nitrogen, stored at −80 °C for total RNA extraction. According to external morphology, color, gonadosomatic index (GSI) and histological feature [15,61], ovarian development was classified into five stages: proliferation (stage I), pre-vitellogenesis (stage II), primary vitellogenesis (stage III), secondary vitellogenesis (stage IV), and tertiary vitellogenesis (stage V). The male crabs were grouped into three stages: spermatocytes (stage I), spermatids (stage II) and mature sperms (stage III). Five crabs at each developmental stage were used for the experiments. All of the study design and animal experiments were conducted in accordance with guidelines of Jimei University’s Animal Care and Use Committee (2011-59).

### 4.2. RNA Isolation and cDNA Synthesis

Total RNA was isolated from these tissues obtained above as described in the previous study [68], and then treated with RNase-free DNase I at 37 °C for 30 min to eliminate residual DNA contamination. For the first-strand cDNA, 3 μg of total RNA from each sample was reverse transcribed using oligo-dT-adaptor primer and M-MLV reverse transcriptase (Promega, Madison, WI, USA) at 42 °C for 90 min.

### 4.3. cDNA Cloning for Sp-uchl3 and Sp-uchl5

Based on the transcriptomics database from our laboratory, the partial cDNA sequences of *Sp-uchl3* and *Sp-uchl5* were obtained. The missing 5′ and 3′ sequences of *Sp-uchl3* and *Sp-uchl5* were obtained using a SMART^TM^-RACE cDNA amplification kit according to the protocol recommended by the manufacturer (BD Biosciences Clontech, Palo Alto, CA, USA). The amplified fragments were resolved by electrophoresis on the agarose gel, and were purified with a gel extraction kit (Generay, Shanghai, China), then inserted into pMD-19T vector (Takara, Beijing, China), propagated in *E. coli* (JM109) competent cells and sequenced. The open reading frames (ORFs) of *Sp-uchl3* and *Sp-uchl5* were confirmed by head-to-toe PCR with three different cDNA templates. We have designed the RACE primers of target genes according to the principle of SMART^TM^-RACE cDNA amplification. All the primers used in this experiment are listed in Table 1.

### 4.4. Bioinformatics Analysis of Sequences

To determine identity of these two genes, nucleotides and predicted amino acid sequence data were compiled and aligned with sequences in Genbank by the Basic Local Alignment Search Tool (BLAST) program (NCBI, Bethesda, MD, USA). (http://blast.ncbi.nlm.nih.gov/Blast.cgi). The open reading frames were analyzed with ORF Finder (NCBI, Bethesda, MD, USA) (http://www.ncbi.nlm.nih.gov/projects/gorf/orfig.cgi). The amino acid sequences were submitted to predict the signal sequence with SignalP 3.0 server (Technical University of Denmark, Lyngby, Denmark) (http://www.cbs.dtu.dk/services/SignalP/). The calculation of the isoelectric point and molecular weight prediction were carried out at http://cn.expasy.org/tools/pi_tool.html, cellular localizations were investigated by PSORTII (Brinkman Laboratory at Simon Fraser University, Vancouver, BC, Canada) (http://www.psort.org/) using the k-nearest neighbor (k-NN), and the phosphorylation sites were analyzed with NetPhos 2.0 Server (Technical University of Denmark, Lyngby, Denmark) (http://www.cbs.dtu.dk/services/NetPhos/). Protein multiple alignments were performed with BioEdit (NC State University, Raleigh, NC, USA) (http://www.mbio.ncsu.edu/BioEdit/). Three-dimensional structures were predicted by using swiss-model at SWISS-MODEL (Swiss Institute of Bioinformatics Biozentrum, University of Basel, Basel, Switzerland) (http://swissmodel.expasy.org/). Phylogenic trees were constructed by a neighbor-joining method with molecular evolutionary genetics analysis 4.0 (MEGA 4.0) software (Institute for Genomics and Evolutionary Medicine, Temple University, Philadelphia, PA, USA). 

### 4.5. RT-qPCR

The mRNA distributions of both *Sp-uchl3* and *Sp-uchl5* in various tissues and in different stages of gonad development were analyzed by RT-qPCR, and these reactions were performed in the Applied Biosystems 7500 real-time system (ABI 7500) using SYBR green PCR Master Mix as recommended by the manufacturer (ABI). Primers used for RT-qPCR are listed in Table 1. Three separate individuals at least, in each gonad development stage and tissues distribution, were tested; all samples were repeated in triplicate for RT-qPCR analysis. The data of the expression levels of *Sp-uchl3* and *Sp-uchl5* were calculated using 2^−∆∆*C*t^ that was normalized with 18S rRNA as described previously [13], and expressed as mean and standard error of the mean (SEM) of RQ value stated. Statistical analysis of the normalized CT values was performed with one-way analysis of variance and Student’s *t*-test conducted using Statistical Package for the Social Sciences 13.0 (SPSS 13.0) software (IBM Corporation, New York, NY, USA). A significant difference was accepted at *p* < 0.05, or most significant at *p* < 0.01

## 5. Conclusions

In this paper, we have successfully identified the full-length cDNAs of *Sp-uchl3* and *Sp-uchl5*, and demonstrated that the expression levels of *Sp-uchl3* and *Sp-uchl5* in the ovary were remarkably higher than in other tissues. Further analysis of the expression of these two genes in the gonad development showed that the expression of *Sp-uchl3* was the highest in the O5 and O1 stages, whereas the expression of *Sp-uchl5* in the O5 stage was the highest, and lower in the three stages of testis. These results suggested that the two genes of UCHs may play different roles in gonad development of the crab.

## Figures and Tables

**Table 1:** Oligonucleotide primers used in this study.

Primer	Primer Sequence (5′-3′)	Purpose
*uchl3*-5′ outer	CACAGCAAAGCGAGACTCATCAGG	5′ RACE
*uchl3*-5′ inner	GGCAGCATCAGCAAGAACAGAGTC	
*uchl3*-3′ outer	GCTTGTGGCACGGTGGCTTTG	3′ RACE
*uchl3*-3′ inner	GCGAAGCCCAAGTCAACG	
RT-*uchl3*-F	GCGAAGCCCAAGTCAACG	qRT-PCR
RT-*uchl3*-R	CCACCACAGCAAAGCGAGA	
*uchl5*-5′ outer	GCCGTGGACAGGTTTCAGTGATG	5′ RACE
*uchl5*-5′ inner	CTTCCACCTGCACACCTTTCACA	
*uchl5*-3′ outer	GCAGCCATCAGGTTCTGTCGTTCA	3′ RACE
*uchl5*-3′ inner	GCCCACATGAAGGGACTTGCTC	
RT-*uchl5*-F	GCCATCAGGTTCTGTCGTTC	RT-qPCR
RT-*uchl5*-R	CATGTGGGCGTCAAAAGTCT	
RT-18S-F	ATGATAGGGATTGGGGTTTGC	RT-qPCR
RT-18S-R	AAGAGTGCCAGTCCGAAGG	
